# Becoming the kind of doctor that you want to be. A qualitative study about participation in Balint group work

**DOI:** 10.1177/00912174211042972

**Published:** 2021-08-30

**Authors:** Elsa Lena Ryding, Anders Birr

**Affiliations:** 1 Department of Women’s and Children’s Health, Institution of Obstetrics and Gynecology, Karolinska Institutet, Sweden; 2 Diagnostic Center, Helsingborg Hospital, Sweden

**Keywords:** Balint group, professional identity, doctor–patient relationship, qualitative research

## Abstract

**Objective:**

Although wide-spread and appreciated, the benefit of Balint group work has been difficult to determine. Qualitative studies provide new angles for research. The aim of this study is to explore how participants in a Balint group for at least 1.5 years experienced the group work and how they were affected by their participation.

**Method:**

Focus group interviews were conducted with a total of 19 members of four different Balint groups. The participants were experienced residents or younger specialists in general practice as well as from hospital specialities. A thematic analysis was performed.

**Results:**

The main themes that emerged were: Investigating emotions, Development of the physician’s identity as well as Safety in the group and with the leader. The participants reported relief from stress as well as increased ability to understand the emotional side of patient encounters. They struggled to find their identity as doctors and specialists, often gaining a sense of pride in their work and becoming more secure. The group with a certified Balint leader felt like a safe place.

**Conclusions:**

For younger doctors, participation in a Balint group for at least 1.5 years can help them build their professional identity by means of a deeper understanding of doctor-patient relationships. The role of Balint group work in relation to professional identity warrants further study.

Today, there are great demands on both young and more experienced doctors. Our work must be both efficient and well done, while at the same time we are exposed to ethical stress. The severe Covid-19 pandemic over the past year has taken a heavy toll on many colleagues. From time to time, it is necessary to stop and reflect on our experiences.^1^ There are various reflection models in healthcare ‒ the Balint group^1,2^ is one. Balint particularly focuses on learning to understand ourselves as well as our patients by reflecting about the patient-doctor interaction and has a long-term perspective on professional development. Today, Balint groups exist worldwide in more than 25 countries (www.balintinternational.com).

The method was first described in Michael Balint's book “The Doctor, his Patient and the Illness”.^3^ By meeting regularly (typically 90 minutes every second week) with one or two Balint leaders in a fixed group of ideally about eight participants, the group provides a secure place for group members to disclose and reflect on their challenges. The group members may learn to better understand their patients and what happens in the patient-physician encounter.

There are three ground rules: 1. Everything said in the group must be treated as strictly confidential. 2. Everyone should be listened to and everyone’s contribution should be respected. 3. Group work focuses on the professional ‒ not the personal ‒ problems of the participants. A Balint group is not group therapy, although therapeutic benefits might be a side effect.^4^

We know that many doctors who have participated in a Balint group are satisfied,^2^ but have they really benefited from the work in the group and if so, how? A 2015 review of existing studies^5^ provided no conclusive answers. The quantitative studies were (too) small and, in addition, differed from each other. There were indications of increased psychosocial competence and reduced risk of burnout, but only after about 1.5 years of participation. The difficulty of gathering sufficiently large comparable groups was highlighted, as well as the need to identify more appropriate dependent variables. Increasing the understanding of the essence of Balint group work is still necessary, for which qualitative exploratory studies were recommended.^5^ Sternlieb^6^ recently applied the autoethnography method to experiences of Balint group work. The themes that emerged were the role of emotions, the importance of a holding space as well as how Sternlieb himself learned and developed as a result of insights into his own emotional landscape. He suggests focus group discussions in order to explore other group members’ learning experiences, which may differ from his own.

Our study aims to explore how participants in a Balint group over a period of at least 1.5 years experienced the group work and how they were affected by their participation.

## Method

Through a network for Balint leaders, we contacted the participants from four groups. In all 20 doctors consented to participate in the study after verbal and written information. No group had the same leader and they worked in four different Swedish cities. All participants had been in the group for at least 1.5 years and all had, when possible, met every second week for 90 minutes. Four focus group interviews were conducted by the first author (ELR) without the respective leaders. One doctor had to cancel her/his participation because of an emergency operation. Two groups were for general practitioners (GPs) or GP residents, one for hospital doctors and one for a mixed group comprising residents in various hospital specialities and GP residents. The interviews lasted about an hour and were recorded. After four interviews no new data or themes emerged.

The groups were asked the following open questions: How did you experience participating in the Balint group? What was good and what was bad? and Did anything change over time?

Follow-up questions were asked as needed.

The interviews were transcribed verbatim (29,360 words). All personal identifiers were removed or changed so that the participants could not be identified.

The transcripts were read on several occasions by the authors separately. The text was analysed drawing on thematic analysis as described by Braun and Clarke.^7^ Initially the authors individually coded the data as well as identified and reviewed the themes. This was followed by several discussions among all the authors, which resulted in the themes and subthemes presented below. No analytic software was used.

## Results

Three themes were identified: Investigating emotions, Development of the physician’s identity as well as Safety in the group and with the leader. The themes and subthemes are presented in Table 1.

**Table 1.** Experience of Balint group work. Themes and subthemes.

Themes	Investigating emotions	Development of the physician’s identity	Safety in the group and with the leader
Subthemes	Relieved by expressing emotions	Insight into emotional reactions facilitates patient encounters	Protective framework
Subthemes	Analy**s**ing emotions in order to understand	Becoming the kind of doctor that you want to be	Room for different leadership styles
Subthemes		A sustainable working life	

### Investigating emotions

All participants in the study were clear about the fact that Balint group discussions concern the emotional side of the work with a focus on the patient-doctor relationship.

#### Relieved by expressing emotions

Most participants experienced reduced anxiety during or after a Balint group session. When someone else presented a case, they could recognize their own feelings and reactions in similar situations and contribute their ideas. Describing their own case allowed them to share their problem(s) with others. It was possible to reveal “forbidden” feelings and thoughts, such as becoming angry with or feeling reprimanded by the patient or not wanting to see her/him again. They felt calmer and more stable when thinking about the next meeting with the patient.

“… you feel a little bad about some things, but then when I come here and find out a little more about it, somehow it becomes clearer … easier to handle.” (Participant Group 1)

They soon felt calmer when faced with difficult or less successful encounters. “Now I can talk about it in the Balint group.” (Participant Group 4)

In particular, GPs seemed to feel very lonely, with a great need to share their thoughts and feelings. Hospital doctors and GP residents appeared to have more opportunities for such sharing.

“As a GP, the Balint group is even more important than it was during your training. As a resident you have other forms of interaction with colleagues, during courses and so on. The group is actually the only place where I have time to reflect during working hours.” (Participant Group 2)

#### Analysing emotions in order to understand

In all four groups, there were doctors who took the exploration of emotions one step further; they had learned to reflect on their own emotional reactions. They could “taste the feeling and get it back”. They also developed their understanding of the patient's or co-worker’s reactions and became more tolerant and flexible towards others’ diversity of expressions and cognitive styles.

“You open your eyes to the fact that there are so many different ways to react, even as a doctor.” (Participant Group 3)

“That you sort of, well, orient yourself in relation to your own feelings. And this ‘Whose feeling is it?’. That’s extremely exciting.” (Participant Group 4)

### Development of the physician’s identity

Developing their professional identity was a significant issue for most participants who were either younger specialists or 3–5 years into their residency.

#### Insight into emotional reactions facilitates patient encounters

The participants claimed that they increasingly understood that each individual interprets and reacts differently to a situation. They became less obsessed about doing “the right thing”. They could accept what had taken place and learn from it. An increased emotional awareness seemed to make them more secure and thus able to provide a safer meeting place for their patients. The group participants believed their diagnostic ability improved when less mental energy was required to cope with difficult encounters.

“I think that, for my part, the big eye opener has probably been that I’m more curious about the difficult cases. ‘But why has it become like this?’ instead of ‘I cannot solve this!’ …So, that makes the job much more fun when you are actually curious and reflect a little. ‘What fate has this patient had in life? Why this?', instead of' ‘This bastard comes and destroys my day’.” (Participant Group 2)

“I do believe that the security I feel has trickled down to the patients too, sort of.” (Participant Group 1)

“I think I have become a better diagnostician. My thoughts are not as cloudy anymore.” (Participant Group 4.)

#### Becoming the kind of doctor that you want to be

The participating doctors obtained help to understand what kind of doctor they wanted to be and then to become that doctor. They felt proud of their profession.

“You are aware of some colleagues who never reflect on anything … I would like to be a colleague who notices and understands things. Both as a doctor and a colleague. Not a robot.” (Participant Group 3)

“Yes, but what has changed in me is that I have developed my role as a doctor. My identity as a doctor has become clearer… How I want to care for my patients and encounter them. And the consultation and such.” (Participant Group 4)

“I also feel that coming here contributes to a sense of pride in our profession. It becomes so clear when you are here. I also feel happy and strengthened. In some way being part of a group like this defines our profession more clearly.” (Participant Group 2)

#### A sustainable working life

The groups expressed a belief that Balint work might make them more resilient. They wanted to continue using their new skills in the future and discussed how to make their superiors understand the importance of an ongoing Balint group.

“I actually experience a much better quality of life since attending the Balint group! Because I have gained a completely different perspective on the job. It's like taking time to feel in a different way instead of just hurrying on.” (Participant Group 2)

“Sure, it's about a sustainable working life. They [the administration] may not think that they want doctors with self-knowledge, but they do want us to continue to work.” (Participant Group 4)

### Safety in the group and with the leader

A warm and safe atmosphere in the group was essential for everyone. The members of the four groups in the study trusted their leaders. The collective group experience was equally important.

“I feel that I have never been able to learn as much about my profession as I do now since I started attending this group. Because all of a sudden, I take part in the vast knowledge and experience of other colleagues’ medical lives and learn so much from hearing their stories and how they think.” (Participant Group 2)

#### Protective framework

The fairly structured framework of Balint group work was initially perceived by some as somewhat unusual and rigid but quite soon as leading to security. Confidentiality was necessary to enable the participants to open up. In other conversations about patients and treatment, such as at clinical meetings or coffee breaks, they could not expect the same level of concern from their colleagues.

“But I also think that you could not have these discussions during a coffee break even if you had an hour… During coffee when you say something like: ‘hearing that was terribly hard…’ you may get the response: ‘well forget about it, that’s not our bloody business’.” (Participant Group 1)

#### Room for different leadership styles

It seemed that the four group leaders worked in somewhat different ways within the framework. The participants found it interesting to discuss their leaders. They were clearly appreciative but also made some critical remarks about the leadership. The leader should not talk too much but at the same time not be too passive. When several members were absent and the group was small, they liked it when the leader joined in and participated in the group work. They appreciated a leader who could open up areas/raise issues they had not considered before. However, they felt ambivalent towards a leader who provided too many of her/his own interpretations. One leader had a more confrontational style. Such interventions could sometimes hurt but at the same time made group members really question their beliefs and actions.

“It hurts there and then, but in the long run it develops your personality a lot. Stimulating discussions, really.” (Participant, Group 1)

By the end of the interview, two groups began discussing taking more responsibility for the sessions. For instance, one group might tell their leader that they needed more help to stay within the framework and not digress by discussing irrelevant matters. Another group resolved to contradict their leader more often when she/he presented interpretations or opinions with which they did not agree.

A few participants, who had previous experience of a (voluntary) Balint group for students, made an interesting remark about its leader, whose style contrasted sharply with that of the four leaders in the present study. The leader had been very silent, making them feel uncomfortable and scrutinized. They opened up but got nothing back. Unfortunately, we have no information about that particular leader’s background. However, the leaders in our study created a secure place for reflection.

## Discussion

The doctors who had attended a Balint group for 1.5 years or more felt safe with the group and its leader. They were helped to examine their feelings in connection with patient encounters. We believe that the most interesting finding is the potential to develop one’s identity as a doctor and gain pride in the profession, based on being the kind of doctor one would like to be rather than concentrating on salary and academic position. Strengthening one’s professional identity and self-esteem may contribute to a sustainable working life.

The nine GPs interviewed by Kjeldmand^8^ from 2002 – 2003 also considered that their competence in the patient encounter had increased thanks to the Balint group. They experienced greater job satisfaction and energy. Several studies comparing doctors who did or did not participate in a Balint group indicate a possible protective effect against burnout in working life.^9,10^ A few recent qualitative studies have used interviews analysed by means of grounded theory^11^ and a phenomenological approach,^12^ respectively. All participants in these two studies were residents. The Iranian study^11^ was conducted after only seven Balint group sessions, which is probably why the doctors seem to make more mention of being overwhelmed by strong feelings and conflicts with other group members. Our study is more in line with the American study based on 24 months of Balint group experience.^12^ Although analysed and presented in a different way, the results seem similar to ours. The most common theme was “Being the physician the patient needs”, which is akin to our theme “Becoming the kind of doctor that you want to be”. Professional identity in medicine may be defined as “how a doctor thinks of himself or herself as a doctor”.^13^ During the years of residency and the first years as a specialist as well as in times of crisis we often have to redefine our professional role. Here, Balint group work may help.

The effect of Balint group leaders on doctors’ learning was the topic of a multicentre study^14^ using the Balint Group Session Questionnaire. However, we were unable to quantify the learning effects. Our impression was that all four groups had learned a great deal, especially about patient-doctor relationships, despite some differences between the respective leaders, which might be insignificant. As in psychotherapy, it is the commitment of the leader/therapist that matters. More importantly, the four leaders in our study are all certified. Their competence includes group dynamics, cultural awareness (of a doctor’s professional life) and psychological/psychotherapeutic competence.

We cannot claim that the Balint method is better for professional development than any other method. As one participant wisely remarked: “(I don’t know) if it's Balint or if it’s that I now have a year and a half more experience.” (Participant Group 1) We can only state that doctors who remained in the group for at least 1.5 years considered that they had learned a great deal about themselves and their professional relationships. Our results may even suggest that the participants have become better doctors, since they tell us about “security (that) has trickled down to the patients too”, being “a better diagnostician”, “more curious about the difficult cases”, and wanting “to be a (doctor) who notices and understands things”.

In our study, no doctor reported any one-sided negative experience. Loyalty towards their leaders and the whole idea of Balint groups may have made them reluctant to express negative critique, even though the interviewer had no connection with the respective groups or workplaces. Nevertheless, it is reasonable to assume that those who stay in the group for three semesters will thrive. However, we cannot exclude that the group members that did not volunteer to participate were less satisfied. It is well known that the Balint method does not suit everyone.^3^ Participation requires a certain mental stability and an open mind. There is a risk that a participant who does not fit in will suffer or at least have a negative experience.^15^ In terms of leadership, there is a fine balance between creating a pleasant environment where no development takes place and challenging (or failing to protect) group members to an extent that makes them close up or quit. In our study, the results support the robustness of the Balint process despite some discomfort among group members.

We assume that other themes may have emerged, had we interviewed groups where all members had attended the group for a longer period or if more participants had been older, more experienced doctors. In a previous study of a Balint group project for anaesthetists, young and very experienced colleagues were mixed in the groups. A theme discussed was the breaking of hierarchy barriers.^16^ Another frequent topic was the difficulties attending experienced by hospital doctors. This was only mentioned in one of the groups in our study, which was for doctors in a surgical speciality.

The concept of professional identity has mainly been studied in students in medical schools and other forms of education.^17^ It could therefore be interesting to investigate the concept in connection with studies of Balint group work in trained physicians.

In conclusion, doctors who wish or are motivated to join a Balint group are likely to gain professionally by their participation. This seems to be a good way to develop a secure professional identity.

Even a virtual Balint group is possible.^18,19^ Ideally, the group members should travel to an initial face-to-face meeting. This mode of working could be useful during times of isolation due to a pandemic as well as in remote areas.
